# A Computational Workflow Translates a 58-Gene Signature to a Formalin-Fixed, Paraffin-Embedded Sample-Based Companion Diagnostic for Personalized Treatment of the BRAF-Mutation-Like Subtype of Colorectal Cancers

**DOI:** 10.3390/ht6040016

**Published:** 2017-11-06

**Authors:** Sjors G. J. G. In’t Veld, Kim N. Duong, Mireille Snel, Anke Witteveen, Inès J. Beumer, Leonie J. M. J. Delahaye, Diederik Wehkamp, René Bernards, Annuska M. Glas, Sun Tian

**Affiliations:** 1 Department of Neurosurgery, VU University Medical Center, 1081HV Amsterdam, The Netherlands; g.intveld1@vumc.nl; 2 Agendia NV, Science Park 406, 1098XH Amsterdam, The Netherlands; kim.duong@agendia.com (K.N.D.); mireille.snel@agendia.com (M.S.); anke.witteveen@agendia.com (A.W.); ines.beumer@agendia.com (I.J.B.); leonie.delahaye@agendia.com (L.J.M.J.D.); diederik.wehkamp@agendia.com (D.W.); r.bernards@nki.nl (R.B.); annuska.glas@agendia.com (A.M.G.); 3 Division of Molecular Carcinogenesis, The Netherlands Cancer Institute, 1066CX Amsterdam, The Netherlands

**Keywords:** bioinformatics workflow, personalized medicine, companion diagnostic, *BRAF*-mutation-like subtype, colorectal cancer, drug repurposing

## Abstract

Colorectal cancer patients with the *BRAF*(p.V600E) mutation have poor prognosis in metastatic setting. Personalized treatment options and companion diagnostics are needed to better treat these patients. Previously, we developed a 58-gene signature to characterize the distinct gene expression pattern of *BRAF*-mutation-like subtype (accuracy 91.1%). Further experiments repurposed drug Vinorelbine as specifically lethal to this *BRAF*-mutation-like subtype. The aim of this study is to translate this 58-gene signature from a research setting to a robust companion diagnostic that can use formalin-fixed, paraffin-embedded (FFPE) samples to select patients with the *BRAF*-mutation-like subtype. *BRAF* mutation and gene expression data of 302 FFPE samples were measured (mutants = 57, wild-type = 245). The performance of the 58-gene signature in FFPE samples showed a high sensitivity of 89.5%. In the identified *BRAF*-mutation-like subtype group, 50% of tumours were known *BRAF* mutants, and 50% were *BRAF* wild-type. The stability of the 58-gene signature in FFPE samples was evaluated by two control samples over 40 independent experiments. The standard deviations (SD) were within the predefined criteria (control 1: SD = 0.091, SD/Range = 3.0%; control 2: SD = 0.169, SD/Range = 5.5%). The fresh frozen version and translated FFPE version of this 58-gene signature were compared using 170 paired fresh frozen and FFPE samples and the result showed high consistency (agreement = 99.3%). In conclusion, we translated this 58-gene signature to a robust companion diagnostic that can use FFPE samples.

## 1. Introduction

The *BRAF*(p.V600E) mutation is found in approximately 10% of colorectal cancer patients [1]. The prognosis of metastatic *BRAF*-mutated tumours is poor [2,3,4]. Colorectal cancer patients with the *BRAF* mutation in their tumours show resistance to the Epidermal growth factor receptor (EGFR) inhibitor Cetuximab and their response to the *BRAF* inhibitor Vemurafenib is limited [5,6]. Personalized treatment options and companion diagnostics are needed to better treat these patients. In order to gain a better understanding of molecular characteristics of these patients and design a personalized treatment for them, we have previously developed a 58-gene signature that characterizes the gene expression pattern of *BRAF*-mutated colorectal tumors. Colorectal tumours identified by this 58-gene signature are called the *BRAF* mutation-like subtype [7]. This subtype represents approximately 20% of colorectal cancers patients, and they include both *BRAF*-mutated tumours and *BRAF* wild-type tumors that share a similar gene expression pattern; therefore, sequencing the *BRAF* mutation alone is not sufficient to capture the whole population of the *BRAF*- mutation-like subtype.

The *BRAF* mutation-like subtype, in both *BRAF* mutated and wild-type forms, displays similar clinical characteristics to *BRAF*-mutated tumors: poor prognosis in a metastatic setting and a tendency towards resistance to anti-EGFR drugs such as Cetuximab [3,4,7,8]. To identify effective therapeutic targets of this specific subtype, 363 upregulated genes of the *BRAF* mutation-like subtype were selected and an RNAi (RNA interference) screen was performed. Knockdown of the *RANBP2* gene led to a defect in microtubule dynamics and apoptosis of *BRAF* mutation-like subtype cell lines during mitosis. Further, both in vitro and in vivo experiments repurposed that the mitotic microtubule-disturbing drug Vinorelbine has the same effect and is specifically lethal to this *BRAF* -mutation-like subtype [9]. These results suggested the possibility of using this 58-gene signature as a companion diagnostic to select patients with the *BRAF*-mutation-like subtype of colorectal cancer to receive Vinorelbine treatment.

The original 58-gene *BRAF*-mutation-like signature was developed by using fresh frozen tissues. To convert it to a companion diagnostic that can be used in clinical practice, the 58-gene signature needs to work with formalin-fixed, paraffin-embedded (FFPE) samples. Formalin fixation and paraffin embedding is the standard storage method in current clinical routine practice by pathologists; a companion diagnostic that can work with FFPE samples will make the logistics of a molecular diagnostic lab practical [10]. However, FFPE samples often produce degraded mRNA and thereby introduce noise into gene expression data, and this introduces a level of complexity [11]. Translation of the 58-gene *BRAF*-mutation-like signature to FFPE demanded two major tasks: (1) the performance of the 58-gene signature on FFPE samples needs to be comparable to the original performance of the 58-gene signature on fresh frozen samples. (2) the readout of the 58-gene signature on FFPE samples need to be robust, and repeated measurements of a companion diagnostic need to show small variation. In this report, we demonstrate a four-steps workflow that Agendia used to convert this 58-gene signature from a research tool that works with fresh frozen samples to a companion diagnostic that works with FFPE samples. First, an FFPE sample set enriched for *BRAF*(p.V600E) mutated tumors was collected. Second, the *BRAF* mutation-like 58-gene signature was translated to FFPE templates and performance was evaluated. Third, the technical stability and reproducibility of the translated FFPE version of the *BRAF* mutation-like 58-gene signature were accessed by using technical control samples. Finally, the borderline region was determined, and the outcome of the translated FFPE version of the *BRAF* mutation-like 58-gene signature was compared to the outcome of the fresh frozen version of the signature by using paired fresh frozen and FFPE samples. Because most molecular signatures were developed by using fresh frozen samples, and most of them were not translated to companion diagnostics that can be used as predictive biomarkers in real clinical practice, the workflow we describe here and alternative approaches presented in the discussion section could serve as an option for scientists who are interested in translating their research results into clinical tools for personalized medicine.

## 2. Materials and Methods

### 2.1. Patients

A total of 302 FFPE colorectal tumor samples were collected and were enriched for *BRAF* mutations. 13 patients had stage I, 218 patients stage II, 69 patients had stage III, and 2 patients had stage IV. The main patient characteristics are depicted in Table 1. All tissue samples were collected from patients with appropriate informed consent. The study was carried out in accordance with the ethical standards of the Helsinki Declaration and was approved by the Medical Ethical Board of the participating medical centers and hospitals in 2011.

### 2.2. Pre-Screen of FFPE Samples Used for Mutational Analysis and Enrichment of BRAF(p.V600E) Mutated Tumor Samples

In order to enrich the *BRAF* mutation samples in the dataset, a pre-screening procedure of FFPE sample selection was performed. The scores of the original *BRAF* mutation-like 58-gene signature for fresh frozen tissues [7] was generated for 602 FFPE samples for which gene expression data were already available, and these 602 FFPE samples were ranked according to their scores. Although these scores were not specifically tailored for FFPE samples, it provided an estimation which samples likely to harbor *BRAF* mutation. The top ranking 96 samples, in addition to a random set of 206 samples were selected for sequencing *BRAF* mutation.

### 2.3. Mutational Analysis

Mutations analysis in *BRAF*(p.V600E) of 206 FFPE samples were assessed in cDNA by Sanger sequencing of PCR products using primers with M13 tails after RT-PCR. *BRAF*(p.V600E) mutations were analysed after amplification of exon 15 using primers 5′-TGATCAAACTTATAGATATTGCACGA (upstream) and 5′-TCATACAGAACAATTCCAAATGC (downstream) [7]. For the remaining 96 FFPE samples, the *BRAF*(p.V600E) were measured with reverse transcription-polymerase chain reaction (RT-PCR) using the QIAGEN therascreen *BRAF* RGQ PCR Kit (Hilden, Germany).

### 2.4. Gene Expression Profiling

All FFPE samples contained at least 30% tumor cells (Supplementary Table S1). RNA isolation, amplification, labelling, hybridization to Agilent full-genome microarrays and data processing of FFPE samples was performed as previous described [10]. RNA extraction was performed using two sections of 10-μm thickness or four sections of 5-μm thickness. Deparaffinization and total RNA extraction was performed using an RNeasy FFPE kit (Qiagen) according to the manufacturer’s instructions. RNA yield was quantified using a NanoDrop spectrophotometer (Thermo Fisher Scientific, Waltham, MA, USA) as described previously [12]. Extracted RNA was amplified using a TransPLEX C-WTA whole-transcriptome amplification kit (Rubicon Genomics, Ann Arbor, MI, USA). Amplified cDNA was labeled using the Genomic DNA Enzymatic Labeling Kit (Agilent Technologies, Santa Clara, CA, USA) and hybridized onto Agendia’s full genome arrays (custom-designed and produced by Agilent Technologies specifically for Agendia), both according to the manufacturer’s instructions. For FFPE samples, no reference channel was used. Gene expression intensities were normalized using Lowess normalization method implemented in Matlab software version R2012a (MathWorks, Inc., Natick, MA, USA).

### 2.5. Translating the Signature to FFPE Templates and Performance of the BRAF Mutation-Like 58-Gene Signature in FFPE Samples

The performance of the 58-gene signature (Supplementary Table S2) in 302 FFPE samples was evaluated by a leave-one-out cross-validation (LOOCV) strategy. For each LOOCV iteration, 1 sample was excluded, and the remaining 301 samples were used to create a new *BRAF* mutation-like template BRAFmutation and a new *BRAF*-WT-like template BRAFwildtype of these 58 genes. A score for the left-out sample was calculated using a nearest centroid classification method with BRAFmutation template and BRAFwildtype template [10]. The threshold was set according to the optimal overall accuracy. A tumor with a score greater than the threshold is classified as the *BRAF*-mutation-like subtype.

### 2.6. Assessment of Stability of the BRAF Mutation-Like 58-Gene Signature in FFPE Samples

Two colorectal tumor samples, one *BRAF*-mutated tumor and one *BRAF* wild-type tumor, were used as technical control samples to evaluate the stability of the *BRAF* 58-gene signature results on FFPE samples. The complete laboratory process including RNA isolation, amplification, labelling, hybridization to Agilent full-genome microarrays, normalization and calculation of *BRAF* 58-gene signature scores of each sample was repeated twice a day over a time period of 20 days. Then, two standard deviations of these two sets of 40 *BRAF* 58-gene signature scores were calculated: one standard deviation for *BRAF* mutated tumors and one standard deviation for *BRAF* wild-type tumors. The range of scores of the *BRAF* 58-gene signature was estimated by using the maximum of the absolute values of signature scores of all 302 FFPE samples (Equation (1)). Stability of the signature was assessed as standard deviations of the control samples divided by the range.

(1) range=[−1×max(abs(set of 302 scores)), max(abs(set of 302 scores))]

## 3. Results

### 3.1. Pre-Screen of FFPE Samples and Mutational Analysis

For the translation of the 58-gene *BRAF*-mutation-like signature from a research tool that works with fresh frozen samples to a companion diagnostic that works with FFPE samples we enriched a gene expression dataset of FFPE tissues with *BRAF* mutated tumors. To have an accurate estimation of sensitivity and specificity of the signature on FFPE samples, the sample size of *BRAF* mutated tumors and *BRAF* wild-type tumors needs to be sufficiently large, e.g., at least greater than 50. The *BRAF* mutation is found in approximately 10% of colorectal cancer patients, as the consequence, the sample size of *BRAF* wild-type tumors is often sufficient, while the sample size of *BRAF* mutated tumors is often much smaller. This low percentage ~10% means that if we aim to collect at least 50 *BRAF* mutation samples by sequencing a random population, the total number of colorectal cancer samples needs to be sequenced in the lab would be more than 500. In order to enrich the *BRAF* mutation samples in the dataset, we generated the scores of the original fresh frozen version of the *BRAF* mutation-like 58-gene signature on FFPE samples, we use these scores to rank our FFPE samples and pre-screened a set of 96 FFPE colorectal tumors that likely harbors high percentage of *BRAF* mutations. The remaining 206 FFPE colorectal tumors were randomly selected.

*BRAF* mutations of these 302 colorectal tumors were measured: as expected, within the pre-screened 96 tumors, a high percentage 35.4% (*n* = 34) harbored p.V600E mutations, and within the randomly selected 206 tumours, 11.2% (*n* = 23) harboured p.V600E mutations. In total, 57 (18.9%) tumours harboured *BRAF* mutations and 245 (81.1%) were the wild-type. Within these 57 *BRAF* mutated tumours, 16 are microsatellite instable tumors (MSI), 7 are microsatellite stable tumors (MSS) and 34 tumors do not have MSI/MSS status available. The percentage of *BRAF* mutations found in these 302 FFPE samples (18.9%) is higher than expected percentage (10%) of a random population. The pre-screen procedure of mutation analysis indeed increased sample size of *BRAF* mutated samples in our FFPE dataset and facilitate an accurate estimation of sensitivity, but because the pre-screen procedure ranked tumors with scores without knowing which tumor is *BRAF* mutated, it also increased the sample size of *BRAF* wild-type tumors that are true *BRAF* mutation-like subtype in these 302 FFPE samples, which resulted in a biased estimation of reduced specificity.

### 3.2. Translating the Signature to FFPE Templates and Performance of the BRAF Mutation-Like 58-Gene Signature in FFPE Samples

**Figure 1:**
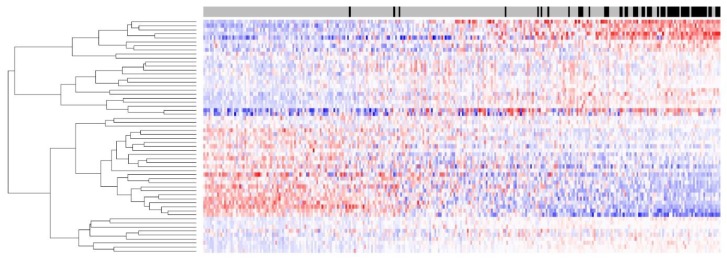
Heatmap of BRAF 58-gene signature, which represents gene expression levels of the signature genes across 302 colorectal tumor samples. Tumors are sorted by their signature scores. Upregulated genes are colored red, and downregulated genes are colored blue. Tumors with known BRAF mutations are displayed as black bars on top of the figure, and tumors without BRAF mutations are displayed as grey bars.

Performance of the *BRAF* mutation-like 58-gene signature was estimated by a leave-one-out cross-validation (LOOCV) on the set of 302 FFPE colorectal tumor samples. Tumors with signature scores above the threshold were assigned to the *BRAF*-mutation-like group, and tumors with signature scores below the threshold were assigned to the wild-type group. The *BRAF* mutation like 58-gene signature predicted 51 of 57 known *BRAF* mutations and predicted 194 of the 245 tumors with no *BRAF* mutation as wild-type (sensitivity 89.5%, specificity 79.2%, Table 2). Figure 1 shows the heatmap of the 58-gene signature on 302 FFPE tumors; the separation of up-/downregulation of 58 genes is clear, indicating that the signal quality for 58 genes derived from FFPE samples was good. Despite the RNA degradation present in this FFPE sample set, a high sensitivity of 89.5% was maintained, and this sensitivity was comparable to that of the same 58-gene signature on a fresh frozen dataset.

The specificity on FFPE sample 79.2% suggests that 20.8% (51 out of 245) of *BRAF* wild-type tumors in this dataset share the same gene expression pattern as *BRAF*-mutated tumours. The specificity 79.2% is lower than the specificity 92% of the 58-gene signature on fresh frozen samples. This is mainly due to the pre-screen procedure used in this study, the top ranking 96 tumours in the pre-screen procedure during the sample collection not only enriched for *BRAF* mutated samples, but also enriched for *BRAF* wild-type samples that are true *BRAF*-mutation-like subtype. The *BRAF*-mutation-like subtype represents approximately 20% of colorectal cancers patients, of which ~10% are known *BRAF* mutated tumors and ~10% are *BRAF* wild-type tumors that share the same gene expression pattern. The total number of the percentage of *BRAF* mutated tumors is enriched from the expected 10% to 18.9% of 302 FFPE samples (57 *BRAF*-mutated tumors), as an estimation, the *BRAF* wild-type tumors in the *BRAF*-mutation-like subtype also should enriched from the expected 10% to 18.9% of 302 FFPE samples (*n* = 57, 302 × 18.9%). Therefore, an expected specificity would be 76.7% (1 − 57/245), and this number is comparable to the observed specificity of 79.2% of the 58-gene signature on 302 FFPE samples. This effect can also be partially inferred by excluding the 96 pre-screened tumor samples. By using only the subset of randomly selected 206 samples to analyze optimal sensitivity (87.0%) and specificity (83.6%), we could observe an increased specificity.

In total, 33.8% of all samples (102 out of 302) were classified as the *BRAF* mutation-like subtype; again, this percentage is high also because of the intentional enrichment for samples with the *BRAF* mutation in this FFPE sample set during sample collection. In the identified *BRAF* mutation-like subtype group, 51 tumors (50%) are known *BRAF*-mutated tumors, and 51 tumors (50%) are *BRAF* wild-types. The exact underlying mechanism why some *BRAF* wild-type tumors display the same gene expression pattern as *BRAF*-mutated tumors is not clear, however, previous pathway analysis performed by our group indicated that mRNA level of activators of MEK/ERK pathway is upregulated and mRNA level of inhibitor of MEK/ERK pathway is downregulated in these *BRAF* wild-types tumors [7]. Taken together, these results indicated that only sequencing the *BRAF* mutation will miss a significant portion of the *BRAF* mutation-like subtype.

### 3.3. Stability of the BRAF Mutation-Like 58-Gene signature in FFPE Samples

**Figure 2:**
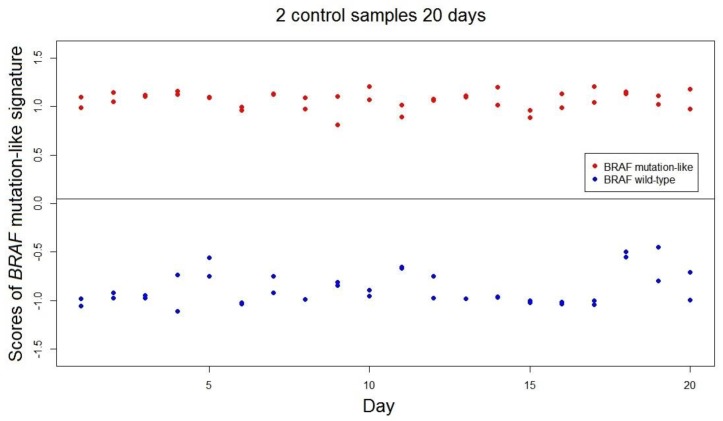
Signature scores of two control samples over 20 days. Controls of *BRAF* mutation-like subtype tumor are displayed as red, and controls of *BRAF* wild-type subtype tumor are displayed as blue. The threshold of the *BRAF* mutation-like 58-gene signature is indicated as the horizontal black line, and no control sample changed outcome during 20 days. The standard deviations of both controls are within the acceptable criteria.

The range of scores of the *BRAF*-mutation-like 58-gene signature was estimated as (−1.53, 1.53; range = 3.06). This value of range is a conservative estimation as the range was estimated by only using 302 samples, and the actual range is expected to be higher than 3.06. The stability and reproducibility over time of the *BRAF* mutation-like 58-gene signature in FFPE samples was measured over 40 independent measurements using 2 FFPE colorectal tumor control samples; one known *BRAF* mutated tumor and one *BRAF* wild-type tumor. All 40 repeated measurements of both controls were correctly classified by the signature. The standard deviations of the scores of *BRAF* mutation-like 58-gene signature are control 1: SD.Brafmut = 0.091, SD.Brafmut/range = 3.0%; control 2: SD.Brafwt = 0.169, SD.Brafwt/range = 5.5% (Figure 2). The empirical predefined criterion is SD/Range not exceeding 10% of the range, and both controls satisfied this criterion [13].

### 3.4. Comparison of the Fresh Frozen Version and the FFPE Version of the BRAF Mutation-Like 58-Gene Signature

**Figure 3:**
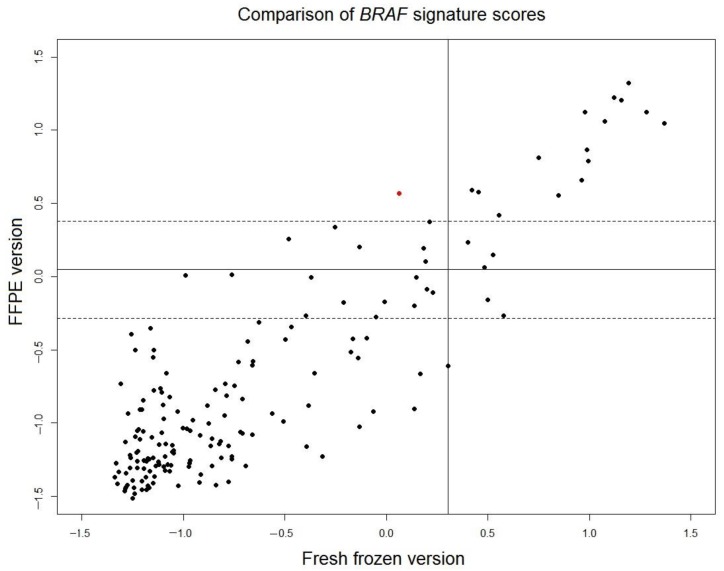
Comparison of scores of the *BRAF* mutation-like 58-gene signature of 170 paired fresh frozen samples and FFPE samples showed that the translated FFPE version is consistent with the fresh frozen version (sensitivity 90%, specificity 92%). The threshold of the fresh frozen version is indicated as the vertical black line and the threshold of the FFPE version is indicated as the horizontal black line. Borderline region of the FFPE version are indicated by horizontal black dash lines. For 148 samples outside of borderline region, only 1 sample (0.7% of total 148 samples), indicated by red, switched outcome between fresh frozen version and FFPE version. The main variation come from two aspects: (1) RNA degradation in FFPE sample, and (2) fresh frozen sample and FFPE sample are two different sampling at two different regions of a same tumor. Scores in the upper right are more correlated than the scores in the lower-left because tumors in the *BRAF* mutation-like subtype (**upper-right**) are more homogenous than tumours that are not in the *BRAF* mutation-like subtypes (**lower-left**), thus the scores changed less by two different sampling.

In the original cohort of fresh frozen samples of 381 tumors used to develop the *BRAF* 58-gene signature [7], paired FFPE samples of 201 tumors were available. Gene expression data of all 201 FFPE samples were measured. Twenty-five FFPE samples with low tumor percentage and six FFPE sample with low signal intensity of microarray were excluded from analysis. The scores of the translated FFPE version of the *BRAF* mutation-like 58-gene signature were read out for the remaining 170 FFPE samples and were compared with the scores of the original fresh frozen version of the signature (Figure 3). The correlation of the scores of FFPE version and fresh frozen version is high (r = 0.88). The borderline region of FFPE scores is defined as threshold±1.96×SD, where sd is set to a conservative estimation as the larger value of standard deviations of two control samples described in the previous section (control 2: SD = 0.169). For 148 samples outside of the borderline region, only 1 sample (0.7% of total 148 samples) switched outcome between fresh frozen version and FFPE version. This result indicated that the outcome of fresh frozen version and translated FFPE version of the *BRAF* mutation-like 58-gene signature is consistent.

## 4. Discussion

The *BRAF* mutation-like subtype of colorectal cancer, including both *BRAF* mutated tumors and *BRAF* wild-type tumors with a similar gene expression pattern, can be characterized by a 58-gene signature. This report described the procedure that translated the 58-gene *BRAF* mutation-like signature developed with fresh frozen tissues to a companion diagnostic that can use FFPE samples. Many molecular signatures have been developed using fresh frozen samples, and translating a molecular signature developed using fresh frozen samples to a companion diagnostic that works with FFPE samples is a critical step in the development of a cancer predictive biomarker that can be used in real clinical practice [10]. Although translating a signature from the fresh frozen setting to the FFPE setting is a technical procedure and logically straightforward, in practice, the advantage and disadvantage of a few alternatives need to be discussed. Generally, there are three different approaches one can choose.

The first approach is to develop a molecular signature directly using FFPE samples. The advantage of this approach that there is no need to process the fresh frozen sample, and it also avoids the possible situation that a molecular diagnostic test developed using fresh frozen samples cannot be translated to a molecular diagnostic test using FFPE samples. The disadvantage of this approach often involves additional studies a molecular diagnostic group wishes to carry out. Often, a molecular diagnostic lab not only aims to develop a molecular diagnostic test that works with FFPE samples but also to identify the best possible genes that characterize prognostic value, drug response or potential intervening targets. For example, in this case, the target gene *RANBP2* was screened by using a fresh frozen data set. To only use FFPE samples would limit the researchers to the degraded mRNA in the FFPE samples and may result in missing the best targets in the discovery phase.

The second approach is to develop a molecular signature using fresh frozen samples first, then translates it using a matching set of fresh frozen and FFPE samples. Here, two samples, one fresh frozen sample and one matching FFPE sample, will be taken from the same tumor, and using this matching dataset, the ideal situation is to observe a linear or adjustable relationship between molecular test scores calculated on fresh frozen and FFPE samples. This approach can identify which individual genes in the molecular signature introduce large variation between fresh frozen samples and FFPE samples. One may consider removing these noise-introducing genes when a molecular signature is translated to the FFPE setting. The disadvantage of this approach comes from the intrinsic noise of both tumor heterogeneity within tumor cells and the mixture of tumor cells and non-neoplastic cells in the tumor tissue. Due to the nature of the process, fresh frozen samples and FFPE samples are taken from different regions of the same tumor. When two different regions of the same tumor are compared, the results are confounded by different mixture of both tumor heterogeneity and presence of non-neoplastic cells. For certain tumor types, for example, colorectal tumors, a tumor sample often contains a diverse mixture of immune cells, stromal cells and tumor cells, and the complexity introduced can be severe.

The third approach is similar to the second one; it develops a molecular signature using fresh frozen samples first, then it does not use a matching set of fresh frozen and FFPE samples, rather, it translates the signature by using a large set of FFPE samples to make new FFPE specific templates and new FFPE specific threshold. The advantage of this approach is that it benefits from the best possible data quality of fresh frozen samples in the discovery phase. The disadvantages are two folds: (1) in some cases, after a molecular diagnostic test developed using fresh frozen samples is translated into one using FFPE samples, its accuracy or stability will decrease too much such that it can no longer be accepted in clinical practice; (2) this approach usually requires an FFPE dataset large enough, often a sample size that is comparable to that of the fresh frozen samples used in the discovery phase.

The workflow described in this report followed the third approach discussed above. In the discovery phase, we benefited from the highest quality data and identified that *RANBP2* knockdown can kill the *BRAF*-mutation-like subtype of colorectal cancer cells [9]. In the companion diagnostic development phase, we showed a robust companion diagnostic that can use FFPE samples to select the *BRAF*-mutation-like subtype. The translated FFPE version of the *BRAF* mutation-like 58-gene signature had a sensitivity of 89.5% and a specificity 79.2%. The specificity of 79.2% is consistent with our expectation because the special pre-screen procedure used in this study enriched percentage of *BRAF* wild-type samples that are true *BRAF*-mutation-like subtype in these 302 FFPE samples, and these 20.8% (51 out of 245) of *BRAF* wild-type tumors are likely true *BRAF* mutation-like subtype. The sensitivity of 89.5% is high and comparable to the sensitivity of fresh frozen version of the signature. A direct evidence of robustness come from the comparison of the signature scores of 148 paired fresh frozen-FFPE samples (Figure 3). The scores of the translated FFPE version of *BRAF*-mutation-like 58-gene signature was compared with the original scores of fresh frozen version (sensitivity 90%, specificity 92%), only 0.7% (1/148) FFPE samples switched outcome. It should be noted that when the 58-gene signature readout was compared between paired fresh frozen samples and FFPE samples, the variation not only comes from RNA degradation in the FFPE sample, but also come from the fact that fresh frozen sample and FFPE sample are essentially two different sampling at two different regions of a same tumor. This variation introduced by two samplings can also be observed in Figure 3. The scores of the translated FFPE version and the fresh frozen version of the tumors in *BRAF* mutation-like subtypes (upper-right, Figure 3) are more consistent than tumors that are not in the *BRAF* mutation-like subtypes (lower-left, Figure 3), because the tumor population of the *BRAF* mutation-like subtype is likely more homogenous than tumors that are not in the *BRAF* mutation-like subtypes. Despite the mix of these two different sources of variations, the comparison showed the performance of the translated FFPE version and the fresh frozen version of the *BRAF* mutation-like 58-gene signature is consistent.

When measuring the sensitivity of a gene signature using mutation statuses, if the threshold of the gene signature is set at the optimal accuracy, a sensitivity of 100% is rarely observed. A few tumor sample may harbor a *BRAF* p.V600E mutation but the dominant gene expression pattern of this tumor sample is not a mutation like. This may be caused by other unknown mechanism that switches off the *BRAF* pathway despite the existence of the *BRAF* mutation, and rarely, the population of the *BRAF* mutated tumor is too small, and the gene expression pattern of mutated tumor cells is blurred by the dominant population of tumor cells of other subtypes in the same tumor sample. For Vinorelbine treatment to show clinical benefit, it is reasonable to assume that the *BRAF* mutation population needs to be dominant in the tumor and resulted in a measurable gene expression pattern, only in this scenario the drug will likely be effective to kill the dominant population of the tumor cells. Thus, the *BRAF*-mutation-like 58-gene signature would be an effective biomarker than sequencing *BRAF* p.V600E alone to select both actual *BRAF* mutation carriers and *BRAF* wild-type in the *BRAF* mutation-like subtype of colorectal cancer patients who may specifically benefit from Vinorelbine treatment. A European Union-funded multicentre clinical trial MoTriColor has started pre-screening for patient enrolment in 2016, and FFPE samples of the primary tumors will be used to test efficacy of Vinorelbine in the *BRAF* mutation-like subtype in this trial.

## Figures and Tables

**Table 1:** Main clinico-pathological data and mutation status of 302 formalin-fixed, paraffin-embedded (FFPE) samples.

	Total Patient Group (*n* = 302) *n* (%)
Sex	
Male	150 (49.7)
Female	152 (50.3)
Age	
Median	70 years
Localisation	
Left	156 (51.7)
Right	140 (46.3)
Unknown	6 (2.0)
T-stage	
1	1 (0.3)
2	16 (5.3)
3	196 (64.9)
4	28 (9.3)
4b	2 (0.7)
Unknown	59 (19.5)
N-status	
0	172 (57.0)
1	44 (14.6)
1b	1 (0.3)
2	18 (6.0)
2a	4 (1.3)
2b	4 (1.3)
Unknown	59 (19.5)
Distal metastasis in follow up	
No	207 (68.5)
Yes	41 (13.6)
Unknown	54 (17.9)
Stage	
1	13 (4.3)
2	218 (72.2)
3	69 (22.8)
4	2 (0.7)
Differentiation Grade	
0	3 (1.0)
0-1	2 (0.7)
1	74 (24.5)
1-2	8 (2.6)
2	148 (49.0)
3	44 (14.6)
Unknown	23 (7.6)
*BRAF* mutation	
Yes	57 (18.9)
No	245 (81.1)
MSI status	
MSI	33 (10.9)
MSS	130 (43.0)
Unknown	139 (46.0)

MSI: Microsatellite instable tumors; MSS: Microsatellite stable tumors.

**Table 2:** Comparison of *BRAF* mutation status and *BRAF*-mutation-like subtype status on 302 FFPE samples.

		*BRAF*(p.V600E) Mutation Status by Sequencing on FFPE Samples	
		Mutated	Wild-type
*BRAF* mutation-like subtype status by 58-gene signature on FFPE samples	*BRAF*-mutation-like	51	51
	*BRAF*-wildtype-like	6	194

## Supplementary Materials

The following are available online at www.mdpi.com/2571-5135/6/4/16/s1: Table S1: Tumor cell percentage and *BRAF* p.V600E mutation status of the FFPE samples; Table S2: List of 58 genes in *BRAF* signature

## References

[1] Tie J., Gibbs P., Lipton L., Christie M., Jorissen R.N., Burgess A.W., Croxford M., Jones I., Langland R., Kosmider S. (2011). Optimizing targeted therapeutic development: Analysis of a colorectal cancer patient population with the BRAF^V600E^ mutation. Int. J. Cancer.

[2] Tol J., Nagtegaal I.D., Punt C.J.A. (2009). BRAF mutation in metastatic colorectal cancer. N. Engl. J. Med..

[3] Bokemeyer C., Bondarenko I., Hartmann J.T., de Braud F., Schuch G., Zubel A., Celik I., Schlichting M., Koralewski P. (2011). Efficacy according to biomarker status of Cetuximab plus FOLFOX-4 as first-line treatment for metastatic colorectal cancer: The OPUS study. Ann. Oncol. Off. J. Eur. Soc. Med. Oncol..

[4] Van Cutsem E., Köhne C.-H., Láng I., Folprecht G., Nowacki M.P., Cascinu S., Shchepotin I., Maurel J., Cunningham D., Tejpar S. (2011). Cetuximab plus irinotecan, fluorouracil, and leucovorin as first-line treatment for metastatic colorectal cancer: Updated analysis of overall survival according to tumor *KRAS* and *BRAF* mutation status. J. Clin. Oncol. Off. J. Am. Soc. Clin. Oncol..

[5] Kopetz S., Desai J., Chan E., Hecht J.R., O’Dwyer P.J., Maru D., Morris V., Janku F., Dasari A., Chung W. (2015). Phase II Pilot Study of Vemurafenib in Patients With Metastatic *BRAF*-Mutated Colorectal Cancer. J. Clin. Oncol. Off. J. Am. Soc. Clin. Oncol..

[6] Pietrantonio F., Petrelli F., Coinu A., Di Bartolomeo M., Borgonovo K., Maggi C., Cabiddu M., Iacovelli R., Bossi I., Lonati V. (2015). Predictive role of *BRAF* mutations in patients with advanced colorectal cancer receiving cetuximab and panitumumab: A meta-analysis. Eur. J. Cancer Oxf. Engl..

[7] Tian S., Simon I., Moreno V., Roepman P., Tabernero J., Snel M., van’t Veer L., Salazar R., Bernards R., Capella G. (2013). A combined oncogenic pathway signature of *BRAF*, *KRAS* and *PI3KCA* mutation improves colorectal cancer classification and cetuximab treatment prediction. Gut.

[8] Popovici V., Budinska E., Tejpar S., Weinrich S., Estrella H., Hodgson G., Cutsem E.V., Xie T., Bosman F.T., Roth A.D. (2012). Identification of a poor-prognosis *BRAF*-mutant-like population of patients with colon cancer. J. Clin. Oncol..

[9] Vecchione L., Gambino V., Raaijmakers J., Schlicker A., Fumagalli A., Russo M., Villanueva A., Beerling E., Bartolini A., Mollevi D.G. (2016). A Vulnerability of a Subset of Colon Cancers with Potential Clinical Utility. Cell.

[10] Sapino A., Roepman P., Linn S.C., Snel M.H.J., Delahaye L.J.M.J., van den Akker J., Glas A.M., Simon I.M., Barth N., de Snoo F.A. (2014). MammaPrint molecular diagnostics on formalin-fixed, paraffin-embedded tissue. J. Mol. Diagn..

[11] Medeiros F., Rigl C.T., Anderson G.G., Becker S.H., Halling K.C. (2007). Tissue handling for genome-wide expression analysis: A review of the issues, evidence, and opportunities. Arch. Pathol. Lab. Med..

[12] Mittempergher L., de Ronde J.J., Nieuwland M., Kerkhoven R.M., Simon I., Rutgers E.J.T., Wessels L.F.A., Van’t Veer L.J. (2011). Gene expression profiles from formalin fixed paraffin embedded breast cancer tissue are largely comparable to fresh frozen matched tissue. PLoS ONE.

[13] Delahaye L.J., Wehkamp D., Floore A.N., Bernards R., van’t Veer L.J., Glas A.M. (2013). Performance characteristics of the MammaPrint breast cancer diagnostic gene signature. Pers. Med..

